# Mortality risk stratification in isolated severe traumatic brain injury using the revised cardiac risk index

**DOI:** 10.1007/s00068-021-01841-7

**Published:** 2021-11-27

**Authors:** Maximilian Peter Forssten, Gary Alan Bass, Kai-Michael Scheufler, Ahmad Mohammad Ismail, Yang Cao, Niels Douglas Martin, Babak Sarani, Shahin Mohseni

**Affiliations:** 1 School of Medical Sciences, Orebro University, 702 81 Örebro, Sweden; 2 Division of Trauma and Emergency Surgery, Orebro University Hospital, 70185 Örebro, Sweden; 3 Division of Traumatology, Surgical Critical Care and Emergency Surgery, University of Pennsylvania, Philadelphia, PA 19104 USA; 4 Department of Neurosurgery, Orebro University Hospital, 70185 Örebro, Sweden; 5 Medical School, Heinrich-Heine University Dusseldorf, Düsseldorf, Germany; 6 Clinical Epidemiology and Biostatistics, School of Medical Sciences, Orebro University, Örebro, Sweden; 7 Division of Trauma and Acute Care Surgery, George Washington University School of Medicine & Health Sciences, Washington, DC USA

**Keywords:** Traumatic brain injury, Revised Cardiac Risk Index, Risk stratification, Mortality

## Abstract

**Purpose:**

Traumatic brain injury (TBI) continues to be a significant cause of mortality and morbidity worldwide. As cardiovascular events are among the most common extracranial causes of death after a severe TBI, the Revised Cardiac Risk Index (RCRI) could potentially aid in the risk stratification of this patient population. This investigation aimed to determine the association between the RCRI and in-hospital deaths among isolated severe TBI patients.

**Methods:**

All adult patients registered in the TQIP database between 2013 and 2017 who suffered an isolated severe TBI, defined as a head AIS ≥ 3 with an AIS ≤ 1 in all other body regions, were included. Patients were excluded if they had a head AIS of 6. The association between different RCRI scores (0, 1, 2, 3, ≥ 4) and in-hospital mortality was analyzed using a Poisson regression model with robust standard errors while adjusting for potential confounders, with RCRI 0 as the reference.

**Results:**

259,399 patients met the study’s inclusion criteria. RCRI 2 was associated with a 6% increase in mortality risk [adjusted IRR (95% CI) 1.06 (1.01–1.12), *p* = 0.027], RCRI 3 was associated with a 17% increased risk of mortality [adjusted IRR (95% CI) 1.17 (1.05–1.31), *p* = 0.004], and RCRI ≥ 4 was associated with a 46% increased risk of in-hospital mortality [adjusted IRR(95% CI) 1.46 (1.11–1.90), *p* = 0.006], compared to RCRI 0.

**Conclusion:**

An elevated RCRI ≥ 2 is significantly associated with an increased risk of in-hospital mortality among patients with an isolated severe traumatic brain injury. The simplicity and bedside applicability of the index makes it an attractive choice for risk stratification in this patient population.

## Introduction

Traumatic brain injury (TBI) continues to be a significant cause of mortality and morbidity worldwide, with at least one-third of all injury-related deaths in the United States carrying a diagnosis of traumatic brain injury [1–7]. Globally, 69 million individuals are estimated to suffer a TBI every year, with approximately 60,000 individuals in the United States and 82,000 individuals in Europe suffering a TBI-related death annually [4, 8, 9]. Particularly among people under the age of 45, TBI is a leading cause of death; however, the highest rates of TBI-related hospitalizations are actually seen among older individuals, particularly those over the age of 75 [4, 6]. In this age group, the rate of TBI-related deaths were estimated to be as high as 77.0 per 100,000 in the United States in 2017, while the corresponding rate was ≤ 15.1 among individuals under the age of 45 [4]. Risk stratification might therefore be an essential tool for the management of this patient population, allowing the early identification of patients with an excess risk of deterioration, death, or other adverse outcomes, and facilitating the distribution of healthcare resources and expertise with greater efficiency. With the contention that cardiovascular events are among the most common extracranial causes of death after a severe TBI, the Revised Cardiac Risk Index (RCRI) may be one contender for this role [10–14].

The RCRI has previously been used to predict the 30-day risk of postoperative myocardial infarction, cardiac arrest, and all-cause mortality [15, 16]. It makes use of only six independent variables: congestive heart failure, ischemic heart disease, cerebrovascular disease, preoperative serum creatinine level above 177 µmol/L (2 mg/dL), diabetes mellitus requiring insulin therapy, and high-risk surgery [15, 16]. Previous studies have demonstrated a clear association between an increase in the RCRI score and excess mortality as well as other adverse outcomes in many non-cardiac surgical procedures [17–21]. Its utility in TBI patients has hitherto not been as thoroughly studied. This investigation aimed to determine the association between the RCRI and in-hospital deaths among isolated severe TBI patients. The hypothesis was that those with an elevated cardiac risk index would exhibit a higher incidence of in-hospital mortality.

## Methods

The need for approval by the institutional review board was waived for this study since it only made use of retrospective anonymized data. The Declaration of Helsinki and the Strengthening the Reporting of Observational Studies in Epidemiology (STROBE) guidelines were adhered to throughout its completion [22]. All data were obtained from the American College of Surgeons Trauma Quality Improvement Program (TQIP) database, a national multi-institutional database of all trauma patients collected from more than 850 participating trauma centers across the United States. This database is gathered for risk-adjusted benchmarking and quality improvement. Furthermore, well-trained data registrars amass more than 100 patient-related and center-related variables. The TQIP plays a central role in the development of evidence-based interventions to enhance patient quality of care by looking at the enrolled centers’ performances [23]. These included patient age, sex, race, initial Glasgow Coma Scale (GCS) in the emergency room (ER), intracranial injuries, abbreviated injury scale (AIS) for each region, surgical interventions, comorbidities, length of stay and in-hospital mortality. All adult patients (18 years or older) registered in the TQIP database between 2013 and 2017 who suffered an isolated severe traumatic brain injury (sTBI) were considered for inclusion. An isolated sTBI was defined as a head AIS ≥ 3 with an AIS ≤ 1 in all other regions. Patients were excluded if they had a head AIS of 6 since these injuries are generally considered non-survivable.

### Calculation of the RCRI

The RCRI was calculated according to Lindenauer et al., which has previously been used in other surgical subspecialties [19–21, 24]. This included the variables congestive heart failure, ischemic heart disease, cerebrovascular disease, renal insufficiency, diabetes mellitus, and high-risk surgery [24]. The RCRI was based on the total number of these factors present in each patient. Both chronic kidney disease and acute kidney injury were included in the definition of renal insufficiency. High-risk surgery was defined as all intraperitoneal, intrathoracic, intracranial, and suprainguinal vascular procedures, according to the 2014 American College of Cardiology/American Heart Association Guideline on Perioperative Cardiovascular Evaluation and Management of Patients Undergoing Noncardiac Surgery [25].

### Statistical analysis

As described by previous studies investigating the RCRI, patients were divided into five cohorts: RCRI 0, RCRI 1, RCRI 2, RCRI 3, and RCRI ≥ 4 [19–21, 24]. Patients’ demographics and clinical features were compared between these cohorts. All continuous variables were presented as a median and interquartile range since they were non-normally distributed. The Kruskall–Wallis test was used to determine the statistical significance of differences between the cohorts. Categorical variables are described as counts and percentages, with comparisons using the Chi-square test or Fisher’s exact test. The primary outcome was in-hospital mortality.

Poisson regression analysis was employed to analyze the association between the RCRI and in-hospital mortality. A Poisson regression model with robust standard errors was used while adjusting for age, sex, race, initial GCS in the ER, AIS in the head, neck, spine, thorax, abdomen, upper extremity, and lower extremity, as well as comorbidities and neurosurgical interventions. Results are presented as an incident rate ratio (IRR) and 95% confidence interval (CI). Multiple imputation by chained equations was used to manage missing values.

Statistical significance was defined as a two-sided *p*-value < 0.05. Analyses were performed using the statistical programming language R (R Foundation for Statistical Computing, Vienna, Austria) [26].

## Results

After applying the inclusion and exclusion criteria, 259,399 patients remained for further analysis. Patients with an elevated RCRI tended to be significantly older (RCRI ≥ 4: 74 years vs RCRI 0: 58 years, *p* < 0.001), were more likely to racially identify as black (RCRI ≥ 4: 15.6% vs RCRI 0: 10.6%, *p* < 0.001), and presented with a higher initial GCS in the ER (RCRI ≥ 4, GCS 14–15: 74.6% vs RCRI 0, GCS 14–15: 69.9%, *p* < 0.001). Accordingly, traumatic subdural hematomas were more prevalent among patients with an elevated RCRI (RCRI ≥ 4: 79.8% vs. RCRI 0: 61.6%, *p* < 0.001), while cerebral contusions and epidural hematomas decreased in prevalence. A higher proportion of patients with an elevated RCRI also underwent a neurosurgical intervention (RCRI ≥ 4 16.8% vs RCRI 0: 12.9%, *p* < 0.001) (Table 1). Nearly all comorbidities increased in prevalence with an increasing RCRI score, which is consistent with expectations and confirms TQIP’s internal validity (Table 2). Length of stay tended to increase with a higher RCRI score (RCRI ≥ 4: 6 days vs. RCRI 0: 3 days, *p* < 0.001). Serial clinically significant increases in crude in-hospital mortality were observed among patients with an increasing RCRI, with an inflection point observed at RCRI ≥ 2 (RCRI ≥ 4: 20.2% vs. RCRI 0: 11.2%, *p* < 0.001) (Table 3).

**Table 1:** Patient demographics and clinical features after an isolated TBI

	RCRI 0 (*N* = 187,442)	RCRI 1 (*N* = 55,792)	RCRI 2 (*N* = 13,346)	RCRI 3 (*N* = 2473)	RCRI ≥ 4 (*N* = 346)	*p*-value
Age, median [IQR]	58 [37–76]	74 [63–82]	75 [67–82]	75 [67–82]	74 [66–81]	< 0.001
Sex, *n* (%)						< 0.001
Female	65,945 (35.2)	22,368 (40.1)	5241 (39.3)	928 (37.5)	131 (37.9)	
Male	121,444 (64.8)	33,408 (59.9)	8104 (60.7)	1545 (62.5)	215 (62.1)	
Missing	53 (0.0)	16 (0.0)	1 (0.0)	0 (0.0)	0 (0.0)	
Race, *n* (%)						
White	142,685 (76.1)	44,011 (78.9)	10,363 (77.6)	1840 (74.4)	255 (73.7)	< 0.001
Black	19,805 (10.6)	5032 (9.0)	1444 (10.8)	344 (13.9)	54 (15.6)	< 0.001
Asian	4646 (2.5)	1784 (3.2)	409 (3.1)	80 (3.2)	8 (2.3)	< 0.001
American Indian	2082 (1.1)	469 (0.8)	93 (0.7)	21 (0.8)	2 (0.6)	< 0.001
Pacific islander	482 (0.3)	158 (0.3)	48 (0.4)	10 (0.4)	1 (0.3)	0.105
Other	13,353 (7.1)	3397 (6.1)	823 (6.2)	151 (6.1)	22 (6.4)	< 0.001
Missing	3720 (2.0)	821 (1.5)	143 (1.1)	22 (0.9)	4 (1.2)	
Initial GCS in the ER, *n* (%)						< 0.001
Mild (GCS 14–15)	130,965 (69.9)	40,792 (73.1)	9624 (72.1)	1774 (71.7)	258 (74.6)	
Moderate (GCS 9–13)	16,676 (8.9)	4991 (8.9)	1306 (9.8)	247 (10.0)	33 (9.5)	
Severe (GCS 3–8)	31,096 (16.6)	6954 (12.5)	1626 (12.2)	314 (12.7)	40 (11.6)	
Missing	8,705 (4.6)	3055 (5.5)	790 (5.9)	138 (5.6)	15 (4.3)	
Intracranial injury, *n* (%)						
Cerebral contusion	46,545 (24.8)	12,080 (21.7)	2735 (20.5)	514 (20.8)	66 (19.1)	< 0.001
Epidural hematoma	9747 (5.2)	1522 (2.7)	353 (2.6)	60 (2.4)	11 (3.2)	< 0.001
Traumatic subdural hematoma	115,452 (61.6)	41,580 (74.5)	10,269 (76.9)	1943 (78.6)	276 (79.8)	< 0.001
Traumatic subarachnoid hemorrhage	55,424 (29.6)	15,877 (28.5)	3494 (26.2)	661 (26.7)	100 (28.9)	< 0.001
Diffuse axonal injury	2478 (1.3)	615 (1.1)	112 (0.8)	16 (0.6)	7 (2.0)	< 0.001
Other intracranial injury	11,358 (6.1)	2214 (4.0)	446 (3.3)	97 (3.9)	18 (5.2)	< 0.001
Head AIS, *n* (%)						< 0.001
3	100,464 (53.6)	26,823 (48.1)	6135 (46.0)	1090 (44.1)	146 (42.2)	
4	47,823 (25.5)	15,583 (27.9)	3601 (27.0)	682 (27.6)	105 (30.3)	
5	39,155 (20.9)	13,386 (24.0)	3610 (27.0)	701 (28.3)	95 (27.5)	
Neck AIS, *n* (%)						< 0.001
Injury not present	185,818 (99.1)	55,478 (99.4)	13,265 (99.4)	2463 (99.6)	344 (99.4)	
1	1624 (0.9)	314 (0.6)	81 (0.6)	10 (0.4)	2 (0.6)	
Thorax AIS, *n* (%)						< 0.001
Injury not present	180,282 (96.2)	53,992 (96.8)	12,943 (97.0)	2403 (97.2)	336 (97.1)	
1	7160 (3.8)	1800 (3.2)	403 (3.0)	70 (2.8)	10 (2.9)	
Abdomen AIS, *n* (%)						< 0.001
Injury not present	183,403 (97.8)	54,849 (98.3)	13,118 (98.3)	2424 (98.0)	331 (95.7)	
1	4039 (2.2)	943 (1.7)	228 (1.7)	49 (2.0)	15 (4.3)	
Spine AIS, *n* (%)						< 0.001
Injury not present	185,107 (98.8)	55,169 (98.9)	13,253 (99.3)	2455 (99.3)	346 (100.0)	
1	2335 (1.2)	623 (1.1)	93 (0.7)	18 (0.7)	0 (0.0)	
Upper extremity AIS, *n* (%)						< 0.001
Injury not present	164,697 (87.9)	49,522 (88.8)	11,897 (89.1)	2219 (89.7)	293 (84.7)	
1	22,745 (12.1)	6270 (11.2)	1449 (10.9)	254 (10.3)	53 (15.3)	
Lower extremity AIS, *n* (%)						< 0.001
Injury not present	170,339 (90.9)	51,126 (91.6)	12,239 (91.7)	2277 (92.1)	313 (90.5)	
1	17,103 (9.1)	4666 (8.4)	1107 (8.3)	196 (7.9)	33 (9.5)	
Neurosurgical intervention, *n* (%)	24,160 (12.9)	8744 (15.7)	2116 (15.9)	444 (18.0)	58 (16.8)	< 0.001
High risk surgery^a^, *n* (%)	0 (0.0)	2794 (5.0)	939 (7.0)	275 (11.1)	54 (15.6)	< 0.001

*TBI* traumatic brain injury, *RCRI* Revised Cardiac Risk Index, *IQR* interquartile range, *GCS* Glasgow Coma Scale, *ER* emergency room, *AIS* abbreviated injury score

^a^High-risk surgery is defined as all intraperitoneal, intrathoracic, and suprainguinal vascular procedures

**Table 2:** Comorbidities in isolated TBI patients

	RCRI 0 (*N* = 187,442)	RCRI 1 (*N* = 55,792)	RCRI 2 (*N* = 13,346)	RCRI 3 (*N* = 2,473)	RCRI ≥ 4 (*N* = 346)	*p*-value
Hypertension, *n* (%)	62,092 (33.1)	39,904 (71.5)	10,518 (78.8)	1993 (80.6)	284 (82.1)	< 0.001
Myocardial infarction, *n* (%)	0 (0.0)	2077 (3.7)	1926 (14.4)	777 (31.4)	211 (61.0)	< 0.001
Congestive heart failure, *n* (%)	0 (0.0)	6082 (10.9)	5465 (40.9)	1753 (70.9)	306 (88.4)	< 0.001
Peripheral vascular disease, *n* (%)	870 (0.5)	860 (1.5)	443 (3.3)	132 (5.3)	27 (7.8)	< 0.001
Cerebrovascular disease, *n* (%)	0 (0.0)	7584 (13.6)	4702 (35.2)	1194 (48.3)	245 (70.8)	< 0.001
Dementia, *n* (%)	11,352 (6.1)	6359 (11.4)	1679 (12.6)	300 (12.1)	41 (11.8)	< 0.001
COPD, *n* (%)	9949 (5.3)	6,007 (10.8)	2,258 (16.9)	493 (19.9)	88 (25.4)	< 0.001
Liver cirrhosis, *n* (%)	2312 (1.2)	1113 (2.0)	283 (2.1)	53 (2.1)	9 (2.6)	< 0.001
Diabetes mellitus, *n* (%)	0 (0.0)	34,774 (62.3)	10,602 (79.4)	2186 (88.4)	332 (96.0)	< 0.001
Coagulopathy, *n* (%)	14,620 (7.8)	11,020 (19.8)	3804 (28.5)	760 (30.7)	111 (32.1)	< 0.001
Chronic kidney disease, *n* (%)	0 (0.0)	1815 (3.3)	2576 (19.3)	1057 (42.7)	223 (64.5)	< 0.001
Acute kidney injury, *n* (%)	0 (0.0)	576 (1.0)	431 (3.2)	153 (6.2)	26 (7.5)	< 0.001
Currently receiving chemotherapy for cancer, *n* (%)	1089 (0.6)	496 (0.9)	94 (0.7)	16 (0.6)	1 (0.3)	< 0.001
Metastatic carcinoma, *n* (%)	1947 (1.0)	1012 (1.8)	243 (1.8)	46 (1.9)	7 (2.0)	< 0.001

*TBI*, traumatic brain injury; *RCRI*, Revised Cardiac Risk Index; *COPD*, chronic obstructive pulmonary disease

**Table 3:** Crude outcomes in isolated TBI patients

	RCRI 0 (*N* = 187,442)	RCRI 1 (*N* = 55,792)	RCRI 2 (*N* = 13,346)	RCRI 3 (*N* = 2473)	RCRI ≥ 4 (*N* = 346)	*p*-value
Length of stay, median [IQR]	3.0 [2.0–6.0]	4.0 [2.0–8.0]	5.0 [3.0–9.0]	5.0 [3.0–11]	6.0 [3.0–13]	< 0.001
Missing, *n* (%)	2966 (1.6)	793 (1.4)	183 (1.4)	43 (1.7)	5 (1.4)	
In-hospital mortality, *n* (%)	20,902 (11.2)	6352 (11.4)	1882 (14.1)	436 (17.6)	70 (20.2)	< 0.001
Missing	26 (0.0)	0 (0.0)	0 (0.0)	0 (0.0)	0 (0.0)	

*TBI* traumatic brain injury, *RCRI* Revised Cardiac Risk Index, *ER* emergency room

After adjusting for potential confounders in the Poisson regression analysis, the same pattern was observed. Compared to patients with RCRI 0, only patients with an RCRI ≥ 2 were observed to have an increased mortality risk. RCRI 2 was associated with a 6% increase in mortality risk [adjusted IRR (95% CI) 1.06 (1.01–1.12), *p* = 0.027], RCRI 3 was associated with a 17% increased risk of mortality [adjusted IRR (95% CI) 1.17 (1.05–1.31), *p* = 0.004], and RCRI ≥ 4 was associated with a 46% increased risk of in-hospital mortality [adjusted IRR (95% CI) 1.46 (1.11–1.90), *p* = 0.006], compared to RCRI 0. RCRI 1 was not associated with an increase in the risk of mortality [adjusted IRR (95% CI) 0.98 (0.95–1.01), *p* = 0.199] (Table 4).

**Table 4:** IRRs for in-hospital mortality after an isolated TBI

	IRR (95% CI)	*p*-value
RCRI		
0	Ref	
1	0.98 (0.95–1.01)	0.199
2	1.06 (1.01–1.12)	0.027
3	1.17 (1.05–1.31)	0.004
≥ 4	1.46 (1.11–1.90)	0.006

Poisson regression model with robust standard errors adjusted for age, sex, race, initial GCS in the ER, AIS, comorbidities, and neurosurgical intervention. Multiple imputation by chained equations was used to manage missing values

*IRR* incident rate ratio, *TBI* traumatic brain injury, *RCRI* Revised Cardiac Risk Index, *GCS* Glasgow Coma Scale, *ER* emergency room, *AIS* abbreviated injury score, *COPD* chronic obstructive pulmonary disease

## Discussion

This is the first study to date investigating the association between the RCRI and in-hospital mortality among patients who suffered an isolated sTBI. As could be observed from crude in-hospital mortality, patients with an RCRI ≥ 2 have an increased risk of in-hospital mortality. The adjusted Poisson regression model confirms this association and further demonstrates that this association remains after adjusting for age, sex, race, initial GCS, severity of the intracranial injury, other underlying comorbidities, and neurosurgical interventions.

An RCRI score of 2 is associated with a 6% increase in the risk of in-hospital mortality, while the risk increases to 17% and 46% at an RCRI score of 3 and ≥ 4, respectively. This threshold is consistent with the American College of Cardiology’s and American Heart Association’s definition of elevated cardiac risk as well as previous research into the utility of the RCRI in patients undergoing an elective resection for colonic tumors [21, 25]. In contrast, previous research into the RCRI’s applicability in hip fractures has demonstrated a stepwise increase in mortality risk for each additional point on the RCRI [18, 19]. This can likely be explained by the high degree of frailty in the hip fracture population due to advanced age and higher prevalence of comorbidities [18, 19, 27–29]. sTBI patients are comparatively younger and have a lower comorbidity burden, as seen in the current data.

Extracranial organ dysfunctions are common among patients with severe TBI, with cardiovascular dysfunction being among the most frequent [10, 12, 13]. Patients with a TBI have been shown to have an almost three-fold increased risk of major adverse cardiovascular events, including heart failure, arrhythmia, myocardial infarction and cardiac necrosis [11]. Accordingly, cardiovascular adverse events are the most common extracranial causes of death in TBI patients [10, 14]. Several studies have shown an association between beta-blocker use and decreased mortality in patients suffering from TBI [30–32], leading to a recommendation for this therapy in this population by the Eastern Association for the Surgery of Trauma [33]. This finding has been attributed to the cardioprotective effect of beta-blockers in response to the hyperadrenergic state often seen in this patient population. In hip fracture patients, where cardiovascular deaths are the most common cause of postoperative mortality [34], an interaction between RCRI and beta-blocker treatment could be noticed with a more pronounced effect of such treatment with an increased RCRI score [35]. Future studies could potentially help identify the subgroup of sTBI patients that, based on their cardiac risk calculated with the RCRI, benefit the most from beta-blockade.

The current study results indicate that TBI patients’ comorbidity burden, as summarized by the RCRI, can be used to stratify patients based on their risk of in-hospital mortality. In the past, other risk scoring systems have been used in the context of TBI patients, including the Charlson Comorbidity Index (CCI) and the APACHE II score [36–40]. Even risk calculators such as Predictive OpTimal Trees in Emergency Surgery Risk (POTTER) could be considered a possible alternative [41, 42]. However, each of these indexes have their limitations. The included variables inherently constrain the APACHE II score, which requires both the results of several blood tests as well as patient vitals, and makes it challenging to apply in the emergency setting [43]. Furthermore, both the CCI and APACHE II require a substantial number of variables, which are incorporated into intricate formulae, to arrive at a final score; this further limits their utility in the acute management of patients [43–45]. POTTER, while demonstrating a substantial predictive ability, also requires the results of several blood tests as well as access to the mobile application to be used [43–45]. The RCRI instead only uses six variables, which can be assessed quickly after a patient arrives in the ER. This simplicity will likely reduce the index’s predictive ability, compared to the other prediction models, but is also key to it being a point-of-care risk stratification tool of some utility in TBI patients.

These characteristics make the RCRI an appealing choice as part of the decision-making process in patients suffering an isolated sTBI. As can be seen in Tables 1 and 2, there is a large degree of heterogeneity among sTBI patients. While the majority of sTBI patients suffer from a relatively low number of comorbidities, those with an elevated RCRI exhibit a significantly higher disease burden, even after excluding the comorbidities included in the RCRI. The proportion of patients with, for example, COPD or a coagulopathy was more than 4 times higher in patients with RCRI ≥ 4 compared to those with RCRI 0. Even the rate of metastatic carcinoma doubled from the lowest RCRI score to the highest. These TBI patients should reasonably be expected to require a different management strategy compared to their healthier counterparts. Using the RCRI, these high risk patients may be identified early on which could allow for a more efficient allocation of expertise and resources [46]. It could also enable the quantification of risk for patients and family members to aid them in participating in the decision making process around critical care, operative interventions, and even goals-of-care [47, 48].

It is also worth mentioning the role of race in the calculation of the RCRI. Previous studies have shown that the traditional formulas for calculating the estimated glomerular filtration rate have tended to underestimate the prevalence of chronic kidney disease in black patients [49–51]. As a result, there is a risk of underestimating the presence of renal insufficiency in this portion of the population. However, in the current study we were able to adjust for race among several other factors. Even after this adjustment, the association between an RCRI score and increased in-hospital mortality remained.

As with all retrospective studies, the current analysis is subject to several limitations. First and foremost, all analyses were restricted to the data available in the TQIP dataset. No adjustments could be made for any potential confounder that was not already registered. The cause of death was also unknown; however, this was of lesser significance as the RCRI includes all-cause mortality among its potential outcomes [16]. This study was also reliant on the accuracy of the data registered in the dataset in order to correctly categorize patients. It is therefore important that the results are interpreted judiciously as this study only provides evidence for an associative relationship between the RCRI and mortality. The RCRI does not include added morbidity or mortality related to intracranial operations and thus may underestimate the overall risk of death in patients with sTBI who require operative intervention. Prospective studies are required to establish a causal relationship, including added morbidity/mortality due to operative intervention, when using the RCRI in reaching treatment decisions. The analysis was also limited to patients with an isolated sTBI, so caution is warranted when considering the results in the context of polytrauma patients with concomitant TBIs. The fact that patients with an RCRI score ≤ 1 still had an in-hospital mortality rate around 11% also indicates that other factors, such as frailty and disease severity, may also need to be considered in order to more fully understand and predict mortality in this patient population, as has been seen when predicting deaths in other traumatic injuries [52].

## Conclusion

An elevated RCRI ≥ 2 is significantly associated with an increased risk of in-hospital mortality among isolated TBI patients. The simplicity and bedside applicability of the index makes it an attractive choice for risk stratification in this patient population. However, further studies are required to determine if there is a causal relationship as well as how it can most effectively be used.
